# The Role of Oral Antibiotic Preparation in Elective Colorectal Surgery: A Meta-analysis

**DOI:** 10.1097/SLA.0000000000003145

**Published:** 2019-07

**Authors:** Katie E. Rollins, Hannah Javanmard-Emamghissi, Austin G. Acheson, Dileep N. Lobo

**Affiliations:** * Gastrointestinal Surgery, Nottingham Digestive Diseases Centre and National Institute for Health Research (NIHR) Nottingham Biomedical Research Centre, Nottingham University Hospitals NHS Trust and University of Nottingham, Queen’s Medical Centre, Nottingham, UK; † MRC/ARUK Centre for Musculoskeletal Ageing Research, School of Life Sciences, University of Nottingham, Queen’s Medical Centre, Nottingham, UK

**Keywords:** anastomotic leak, colorectal, mechanical bowel preparation, oral antibiotics, surgery, surgical site infection

## Abstract

**Objectives:**

To compare the impact of the use of oral antibiotics (OAB) with or without mechanical bowel preparation (MBP) on outcome in elective colorectal surgery.

**Summary Background Data:**

Meta-analyses have demonstrated that MBP does not impact upon postoperative morbidity or mortality, and as such it should not be prescribed routinely. However, recent evidence from large retrospective cohort and database studies has suggested that there may be a role for combined OAB and MBP, or OAB alone in the prevention of surgical site infection (SSI).

**Methods:**

A meta-analysis of randomized controlled trials and cohort studies including adult patients undergoing elective colorectal surgery, receiving OAB with or without MBP was performed. The outcome measures examined were SSI, anastomotic leak, 30-day mortality, overall morbidity, development of ileus, reoperation and *Clostridium difficile* infection.

**Results:**

A total of 40 studies with 69,517 patients (28 randomized controlled trials, n = 6437 and 12 cohort studies, n = 63,080) were included. The combination of MBP+OAB versus MBP alone was associated with a significant reduction in SSI [risk ratio (RR) 0.51, 95% confidence interval (CI) 0.46–0.56, *P* < 0.00001, I^2^ = 13%], anastomotic leak (RR 0.62, 95% CI 0.55–0.70, *P* < 0.00001, I^2^ = 0%), 30-day mortality (RR 0.58, 95% CI 0.44–0.76, *P* < 0.0001, I^2^ = 0%), overall morbidity (RR 0.67, 95% CI 0.63–0.71, *P* < 0.00001, I^2^ = 0%), and development of ileus (RR 0.72, 95% CI 0.52–0.98, *P* = 0.04, I^2^ = 36%), with no difference in *Clostridium difficile* infection rates. When a combination of MBP+OAB was compared with OAB alone, no significant difference was seen in SSI or anastomotic leak rates, but there was a significant reduction in 30-day mortality, and incidence of postoperative ileus with the combination. There is minimal literature available on the comparison between combined MBP+OAB versus no preparation, OAB alone versus no preparation, and OAB versus MBP.

**Conclusions:**

Current evidence suggests a potentially significant role for OAB preparation, either in combination with MBP or alone, in the prevention of postoperative complications in elective colorectal surgery. Further high-quality evidence is required to differentiate between the benefits of combined MBP+OAB or OAB alone.

Surgical site infection (SSI) is a major burden for patients undergoing elective colorectal surgery. It adds significantly to the cost of health care, and administration of preoperative bowel preparation has been proposed to reduce the incidence of SSI. The role of mechanical bowel preparation (MBP) with polyethylene glycol or sodium phosphate has been studied in randomized controlled trials (RCTs), with perceived benefits including ease of manipulation of the bowel, reduced spillage and resultant contamination, reduced luminal pressure, and lesser bacterial load. However, a recent metaanalysis^1^ of 36 RCTs and cohort studies, and an earlier one^2^ of 14 RCTs found that that the administration of MBP did not impact upon postoperative morbidity or mortality. This, in combination with high rates of patient dissatisfaction and fluid and electrolyte disturbances, has led to the conclusion that MBP should not be prescribed routinely. This is reflected in Guidelines from the Enhanced Recovery After Surgery Society,^3^,^4^ the National Institute of Health and Care Excellence,^5^ and the American Society for Enhanced Recovery,^6^ all of which suggest that MBP should not be administered routinely. However, although the American Society for Enhanced Recovery guidelines suggest that MBP should not be given in isolation, they recommend routine use of an isosmotic bowel preparation and combined oral antibiotic prior to elective colorectal surgery.^6^

The use of oral antibiotic (OAB) prophylaxis, in the form of nonabsorbable luminal antibiotics, was first proposed in 1971 by Rosenberg et al^7^ in a RCT of 150 patients undergoing large bowel surgery receiving MBP alone, or MBP in combination with phthalylsulphathiazole or phthalylsulphathiazole and neomycin. The combination of MBP+OAB was associated with a significant reduction in SSI (23% vs. 40%), anastomotic leak rates (24% vs. 52%), and sepsis rates (37.3% vs. 64.4%).^6^ Although several studies provided evidence for the role of oral antibiotics in elective colorectal surgery, the regimens included large volume preparations,^8^–^10^ prolonged preoperative hospital admission, and in the setting of prolonged preoperative starvation protocols, dehydration, and electrolyte disturbances were commonplace.^11^,^12^ Decreased compliance and inconsistent bowel cleansing resulted in a reduced intervention effect and, this, combined with reduced preoperative admission times, resulted in the practice of combined MBP+OAB dwindling in favor of more restrictive MBP regimens alone. However, recently there has been resurgent interest in the use of OAB in colorectal surgery,^13^,^14^ particularly in light of a large number of retrospective cohort and database studies, many of which originated from the American College of Surgeons National Surgical Quality Improvement Program (ACS NSQIP) targeted colectomy database.^15^–^20^ Evidence for the role of OAB has been summarized in several narrative reviews^21^,^22^ as well as meta-analyses,^23^–^25^ which have supported a reduction in SSI associated with combined MBP, OAB, and parenteral antibiotics over MBP and parenteral antibiotics alone. However, the most recent of these studies have been flawed in their inclusion of multiple studies based on the NSQIP database which have large degrees of cross-over of the same study population and have mostly focused upon SSI alone rather than other postoperative outcomes. In addition, recent studies^18^,^26^ have suggested that OAB alone may provide equivalent prophylaxis in terms of SSI and anastomotic leak rates when compared with a combined regimen of MBP+OAB.

The aims of this meta-analysis of RCTs and observational cohort studies in patients undergoing elective colorectal surgery were to:

- Compare the impact of OAB with or without MBP in elective colorectal surgery in terms of SSI, anastomotic leak, 30-day mortality, overall morbidity, development of ileus, reoperations, and *Clostridium difficile* infection.
- Compare evidence derived from RCTs and cohort studies.
- Compare the role of administration of OAB with and without MBP in the setting of laparoscopic versus open surgery.

## Methods

### Search Strategy

The PubMed, Google Scholar, MEDLINE, and the Cochrane Library databases were searched to identify studies evaluating the effect of OAB in adults undergoing elective colorectal surgery published between January 1, 1981 and May 30, 2018. This date restriction was imposed as recommendations that parenteral antibiotics should be administered routinely for prophylaxis against SSI in colorectal surgery were made in 1981^27^ and it was felt that all studies considering the role of oral antibiotic prophylaxis should include parenteral antibiotic prophylaxis, to reflect current perioperative care. The search terms used were: (oral antibiotic OR oral antibacterial) AND (colon OR rectal OR colorectal) AND surgery. The bibliographies of all studies which met the inclusion criteria, and previous systematic reviews and meta-analyses on the subject were reviewed to ensure study inclusion was as complete as possible. Non-English-language papers were translated for inclusion. The meta-analysis was conducted in accordance with the PRISMA statement.^28^

### Selection of Articles

Articles were screened for suitability on the basis of title and abstract by 2 independent researchers (K.E.R. and H.J.-E.). Studies were eligible for inclusion if they examined the role of OAB preparation with or without MBP, compared with either MBP alone, OAB alone, or no preparation in adult patients due to undergo elective colorectal surgery, with at least 1 relevant clinical outcome reported. The type of colorectal surgery performed in terms of type of resection or laparoscopic versus open, the presence or absence of rectal enema administration, or the indication for surgery were not discriminants. Studies were excluded if they did not consider any relevant clinical outcomes, included emergency procedures, or duplicated study populations from other included studies. From the large number of ACS NSQIP studies published^15^–^20^,^26^,^29^–^40^ (Supplementary Table 1, http://links.lww.com/SLA/B542), only the largest study by Midura et al^31^ was included to avoid the risk of duplication of patient populations within the analysis. Similarly, 3 publications^41^–^43^ originated from the Michigan Surgical Quality Collaborative Colectomy Best Practices Project. When these were reviewed, 2 studies^41^,^42^ considered the same comparison of preparations (MBP+OAB vs no preparation), and as such only the more comprehensive study including a larger number of clinical outcomes was included.^41^ The third study from the Michigan Surgical Quality Collaborative database^43^ examined a different preparation combination, thus this was included in the meta-analysis. Finally, the national Veterans Affairs Surgical Quality Improvement Program was the basis for 2 studies^44^,^45^ on the same regimen comparison, thus only the largest study was included within the meta-analysis.^45^ One study^46^ included a small proportion of patients undergoing emergency colorectal resection within the cohort (311 of a total population of 2240), so any outcomes that included this study were analyzed both with and without it included to discern any difference in results.

### Data Extraction

Data were extracted by 2 independent researchers (K.E.R. and H.J.-E.) and any discrepancies were resolved by a senior author (D.N.L.). The primary outcome measure was SSI, with secondary outcome measures including anastomotic leak, 30-day mortality, overall morbidity, development of ileus, reoperation, and *Clostridium difficile* infection. Data were also collected on patient demographics (age, sex), surgical variables (type of resection, open vs. laparoscopic, underlying disease necessitating resection), and details of the preparation used, in terms of parenteral and oral antibiotics as well as MBP. Several studies stated that MBP was not used in patients with obstructing masses, which is mirrored in standard clinical practice, thus these papers were included in the meta-analysis.

The risk of bias was assessed for the RCTs included using the Cochrane Collaboration tool within the RevMan software^47^ which considers random sequence generation (selection bias), allocation concealment (selection bias), blinding of participants and personnel (performance bias), blinding of outcome assessment (detection bias), incomplete outcome data (attrition bias), and selective reporting (reporting bias).

### Statistical Analysis

Data were entered into RevMan 5.3 software.^47^ Dichotomous variables were calculated as risk ratios (RR) with a 95% confidence interval using the Mantel–Haenszel random effects model. From this, forest plots were derived, with a *P* value of less than 0.05 on 2-tailed testing representing a statistically significant difference. Data from RCTs and cohort studies were included separately within each forest plot, with a summative analysis of all the evidence performed in addition. Inconsistency and heterogeneity between studies were estimated using the I^2^ statistic;^48^ ≤25% represented low heterogeneity, 25% to 50% represented moderate, and >50% high heterogeneity.

### Protocol Registration

The protocol for this meta-analysis was registered with the PROSPERO database (www.crd.york.ac.uk/prospero)—registration number CRD42018098950.

## Results

From the 520 studies identified in the initial search, 40 studies^31^,^41^,^43^,^45^,^46^,^49^–^83^ on 69,517 participants were included (Supplementary Figure 1, http://links.lww.com/SLA/B542). Of these 28 were RCTs with 6437 participants^49^–^53^,^55^–^59^,^61^–^67^,^69^–^73^,^75^,^76^,^78^–^80^,^83^ and 12 were cohort (case control) studies with 63,080 participants.^31^,^41^,^43^,^45^,^46^,^54^,^60^,^68^,^74^,^77^,^81^,^82^ The risk of bias in the RCTs included was variable, with poor levels of documentation particularly surrounding randomization methods, allocation concealment, and blinding in the earlier studies (Table 1). Six studies^57^,^58^,^62^,^64^–^66^ administered different parenteral antibiotic regimens depending upon whether the patient was receiving MBP+OAB or MBP alone, which may provide significant source of bias in terms of SSI prevention. In addition, 1 study^73^ included 2 differing parenteral antibiotic regimens, both in combination with MBP, versus OAB, MBP and parenteral antibiotics. As both of the parenteral antibiotic regimens were considered eligible for inclusion, these were grouped together to form the MBP alone group. In terms of oral antibiotics, 2 studies administered OAB preparation only on the day of surgery; one^64^ gave ciprofloxacin 1 g 1 hour preoperatively and the other^74^ ciprofloxacin 750 mg 1 to 3 hour preoperatively. A subgroup of another study^51^ received only 1 dose of OAB the day before surgery, with the remainder receiving 3 doses. These 3 studies may, therefore, have an attenuated the intervention effect from the OAB administered.

### Patient Demographics

Two studies^53^,^55^ focused on surgery using laparoscopic techniques, 21 on open surgery alone,^46^,^50^,^52^,^57^,^58^,^61^,^62^,^64^–^74^,^76^,^78^,^80^ with 9 studies^41^,^43^,^49^,^54^,^60^,^75^,^77^,^81^,^82^ mixing both open and laparoscopic techniques and the remaining 8 studies not providing this information.^31^,^45^,^51^,^56^,^59^,^63^,^79^,^83^ The most recent publication^31^ included patients undergoing robotic surgery. The indication for surgery was colorectal cancer in 8 studies,^46^,^54^,^55^,^59^,^61^,^75^,^78^,^81^ inflammatory bowel disease in 2,^67^,^80^ with the remaining including a mixture of benign and malignant pathologies. Patient demographics and surgical variables as well as the details of MBP, OAB, and parenteral antibiotics administered are detailed in Table 2.

### Surgical Site Infection (SSI)

#### MBP+OAB Versus MBP

**Figure 1:**
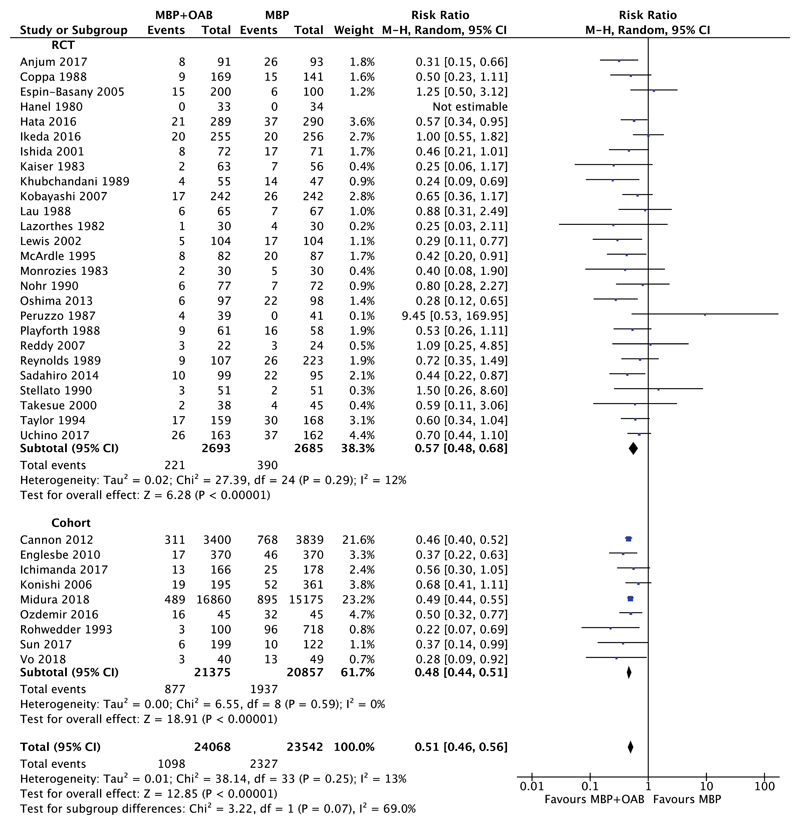
Forest plot comparing surgical site infection rate for patients receiving MBP+OAB versus MBP alone, divided by evidence from RCTs and cohort studies. A Mantel–Haenszel random effects model was used to perform the meta-analysis and risk ratios are quoted including 95% confidence intervals.

The comparison between MBP+OAB versus MBP alone was performed in 35 studies; 26 RCTs^49^–^53^,^55^–^59^,^61^–^67^,^69^,^70^,^72^,^73^,^75^,^76^,^78^–^80^ and 9 cohort studies^31^,^43^,^45^,^54^,^60^,^68^,^74^,^77^,^81^ with a total of 47,610 patients. When all studies were considered (Fig. 1), the combination of MBP+OAB was associated with a significant reduction in SSI versus MBP alone (RR 0.51, 95% CI 0.46–0.56, *P* < 0.00001, I^2^ =13%). The results remained consistent when just RCT studies were examined (5378 patients; RR 0.57, 95% CI 0.48–0.68, *P* < 0.00001, I^2^ = 12%), as well as cohort studies (42,232 patients; RR 0.48, 95% CI 0.44–0.51, *P* < 0.00001, I^2^ = 0%).

#### MBP+OAB Versus OAB

**Figure 2:**
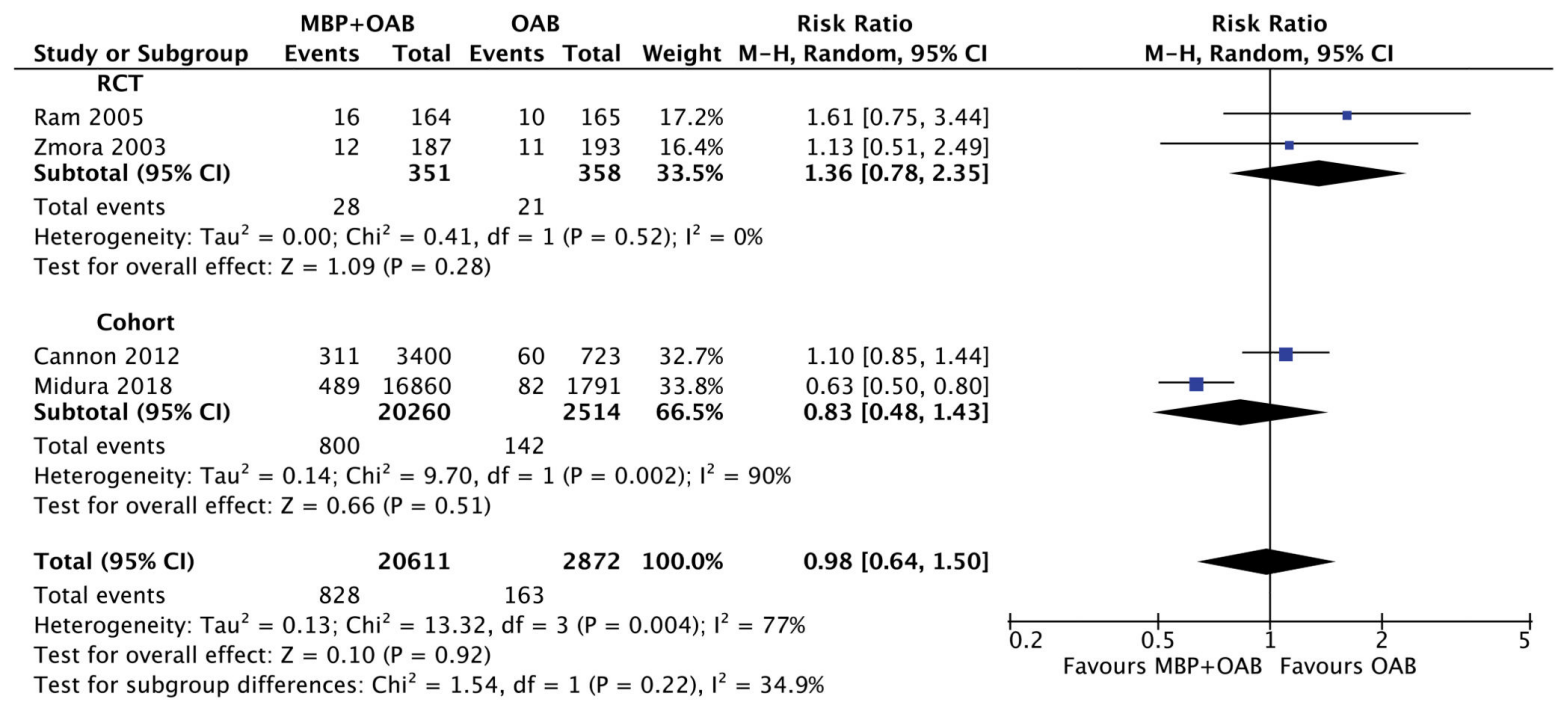
Forest plot comparing surgical site infection rate for patients receiving MBP+OAB versus OAB alone, divided by evidence from RCTs and cohort studies. A Mantel–Haenszel random effects model was used to perform the meta-analysis and risk ratios are quoted including 95% confidence intervals.

The analysis of MBP+OAB versus OAB alone was considered by 4 studies; 2 RCTs^71^,^83^ and 2 cohort studies^31^,^45^ including 23,483 patients (Fig. 2). Overall, the combination of MBP+OAB was not associated with any difference in the incidence of SSI versus OAB alone (RR 0.98, 95% CI 0.64–1.50, *P* = 0.92), with high heterogeneity (I^2^ = 77%). When RCTs alone were considered, again no difference was seen (RR 1.36, 95% CI 0.78–2.35, *P* = 0.28, I^2^ = 0%), as with cohort studies (RR 0.83, 95% CI 0.48–1.43, *P* = 0.51, I^2^ = 90%).

#### MBP+OAB Versus No Preparation

No RCTs considered the comparison between combined MBP+OAB and no preparation, with evidence arising from just 4 cohort studies (36,642 patients).^31^,^41^,^45^,^46^ The combination of MBP+OAB was associated with a significant reduction in SSI (RR 0.54, 95% CI 0.43–0.68, *P* < 0.00001, I^2^ = 82%) when compared with no preparation.

#### OAB Alone Versus No Preparation

No RCTs focused upon the comparison between OAB alone versus no preparation, with evidence arising from 16,390 patients included in 2 cohort studies.^31^,^45^ OAB alone reduced the incidence of SSI versus no preparation (RR 0.56, 95% CI 0.38–0.83, *P* = 0.004, I^2^ = 81%).

#### OAB Versus MBP

Two studies^31^,^45^ considered the incidence of SSI with OAB alone versus MBP alone, with OAB associated with a reduction in SSI rates. However, this did not reach statistical significance (RR 0.57, 95% CI 0.31–1.05, *P* = 0.07, I^2^ = 93%).

### Anastomotic Leak

#### MBP+OAB Versus MBP

**Figure 3:**
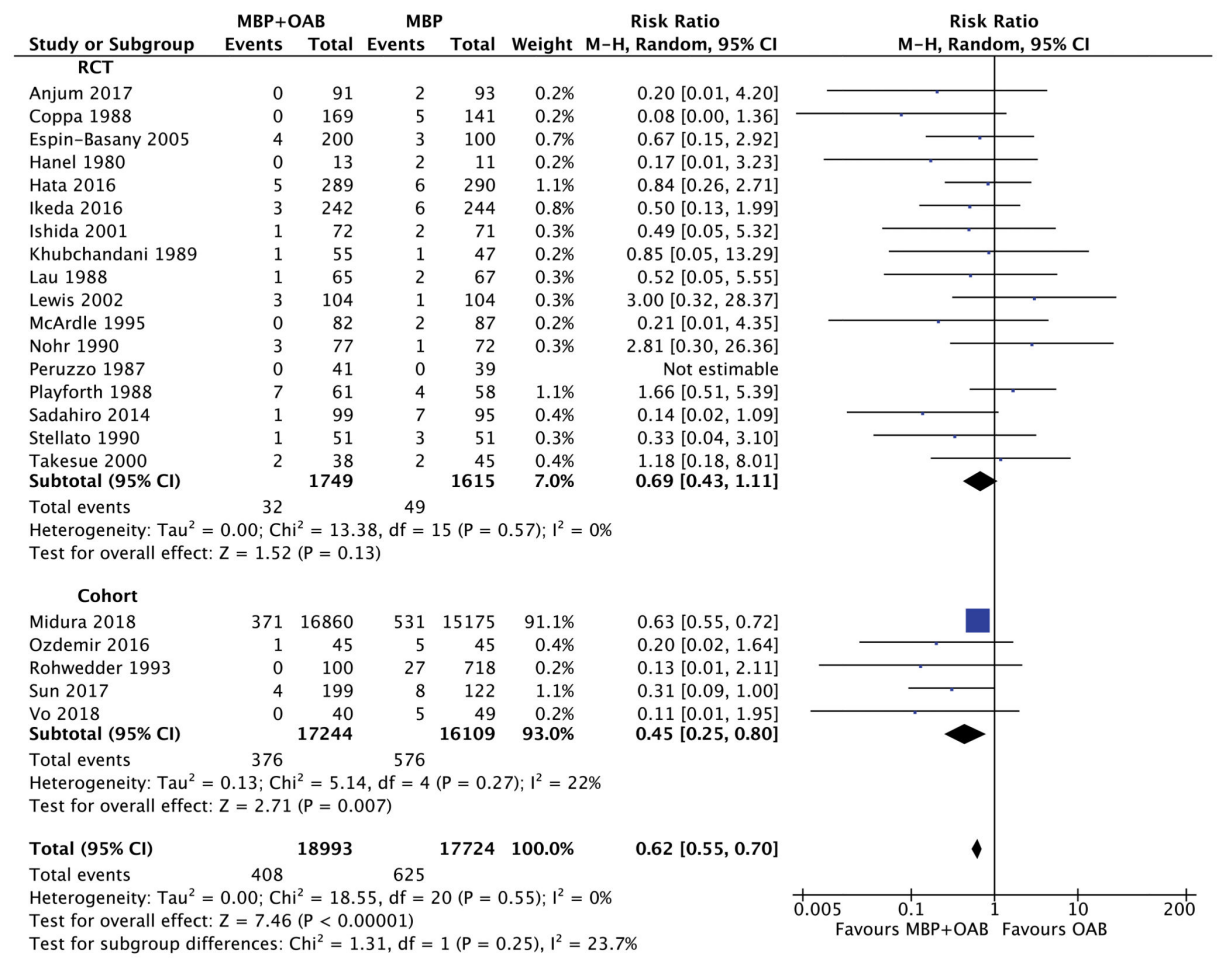
Forest plot comparing anastomotic leak rate for patients receiving MBP+OAB versus MBP alone, divided by evidence from RCTs and cohort studies. A Mantel–Haenszel random effects model was used to perform the meta-analysis and risk ratios are quoted including 95% confidence intervals.

Rates of anastomotic leak in those receiving combined MBP+OAB versus MBP alone were compared in 22 studies (Fig. 3); 17 RCTs^49^–^53^,^55^,^56^,^58^,^61^,^63^,^64^,^66^,^69^,^70^,^75^,^76^,^78^ and 5 cohort studies.^31^,^68^,^74^,^77^,^81^ Only 2 RCTs^49^,^52^ included data regarding the management of the anastomotic leak, with none of the 124 patients receiving combined MBP+OAB requiring return to theater for anastomotic leakage compared with 2 of 127 patients receiving MBP alone. Overall, the combination of MBP+OAB was associated with a significant reduction in anastomotic leak rates (RR 0.62, 95% CI 0.55–0.70, *P* < 0.00001, I^2^ = 0%), and when evidence from cohort studies alone was considered (RR 0.45, 95% CI 0.25–0.80, *P* = 0.007, I^2^ = 22%), but no significant difference was seen when RCTs were analyzed (RR 0.69, 95% CI 0.43–1.11, *P* = 0.13, I^2^ = 0%). Six studies^51^,^53^,^55^,^68^,^77^,^81^ included data on the use of a diverting stoma, with 133 patients of 1028 in the combined MBP+OAB group and 99 patients of 862 in the MBP alone group undergoing a protective stoma formation.

#### MBP+OAB Versus OAB

The combination of MBP+OAB versus OAB alone was considered by 3 studies; 2 RCTs^71^,^83^ and 1 cohort study,^31^ with no difference observed in anastomotic leak rates when all studies (RR 0.79, 95% CI 0.59–1.05, *P* = 0.11, I^2^ = 0%), or just RCTs (RR 1.39, 95% CI 0.47–4.10, *P* = 0.55, I^2^ = 0%) were considered (Supplementary Figure 2, http://links.lww.com/SLA/B542). No data were available on return to theater rates related to anastomotic leaks.

#### MBP+OAB Versus No Preparation

The comparison between MBP+OAB versus no preparation in terms of anastomotic leak was considered by just 2 cohort studies,^31^,^46^ with combined MBP+OAB being associated with a significant reduction in anastomotic leak rates (RR 0.52, 95% CI 0.45–0.59, *P* < 0.00001, I^2^ = 0%). No data were available on return to theater rates secondary to anastomotic leaks or diverting stoma rates.

#### Other Comparisons

The comparison of anastomotic leak rates between OAB alone versus no preparation and OAB versus MBP was each only considered by 1 cohort study,^31^ and as such meta-analysis was not feasible.

### 30-day Mortality

#### MBP+OAB Versus MBP

**Figure 4:**
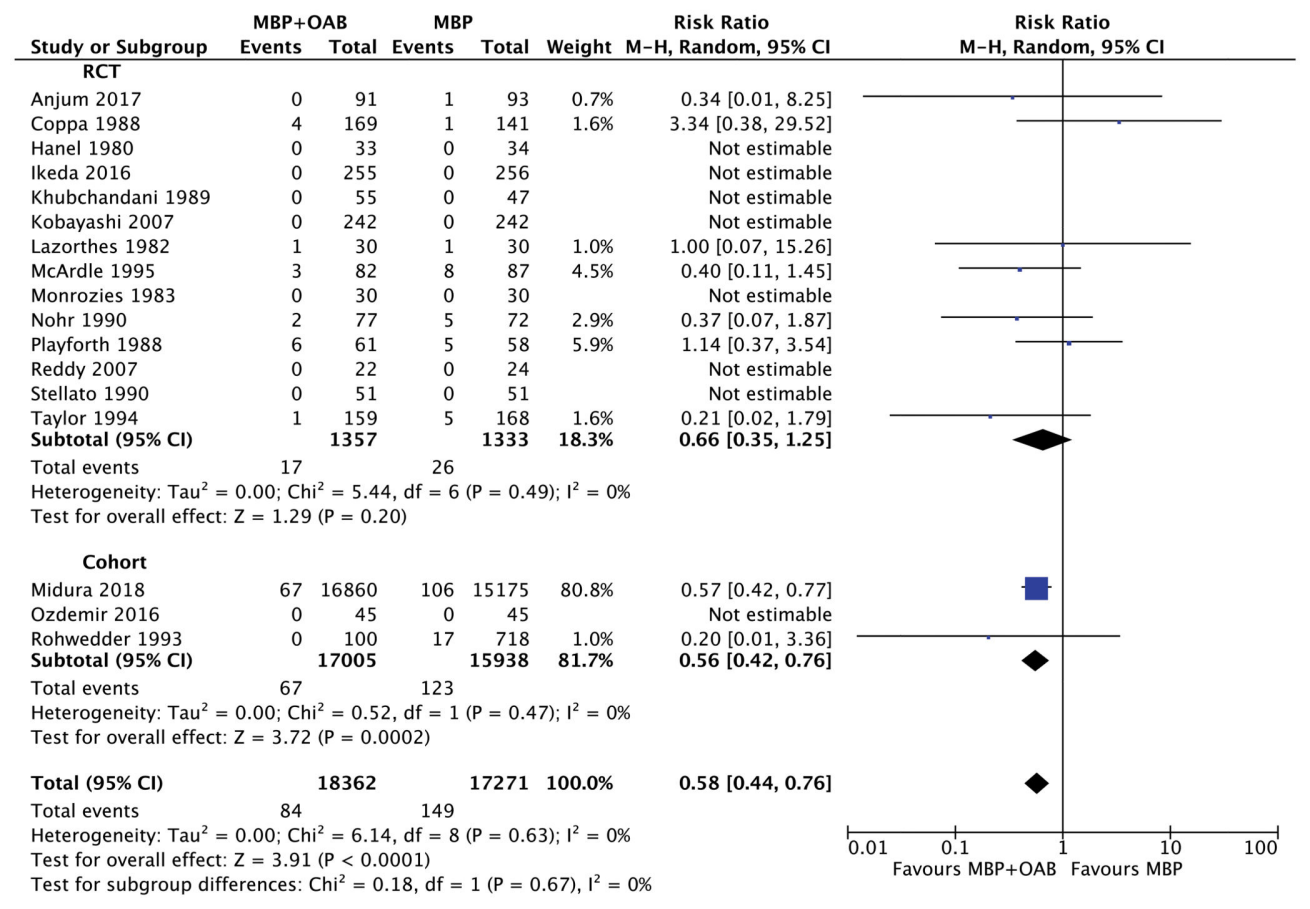
Forest plot comparing 30-day mortality rates for patients receiving MBP+OAB versus MBP alone, divided by evidence from RCTs and cohort studies. A Mantel–Haenszel random effects model was used to perform the meta-analysis and risk ratios are quoted including 95% confidence intervals.

Seventeen studies (35,633 patients) examined 30-day mortality rates between those receiving MBP+OAB versus MBP alone; 14 RCTs^49^,^50^,^52^,^55^,^58^,^59^,^62^,^64^–^66^,^70^,^72^,^76^,^79^ and 3 cohort studies^31^,^68^,^74^ (Fig. 4). Overall, the combination of MBP+OAB was associated with a significant reduction in 30-day mortality versus MBP alone (RR 0.58, 95% CI 0.44–0.76, *P* < 0.0001, I^2^ = 0%). This was also the case when evidence arising from cohort studies alone was considered (RR 0.56, 95% CI 0.42–0.76, *P* = 0.0002, I^2^ = 0%), but not when RCTs alone were examined (RR 0.66, 95% CI 0.35–1.25, *P* = 0.20, I^2^ = 0%).

#### MBP+OAB Versus OAB

Three studies (2 RCTs^71^,^83^ and 1 cohort study^31^) including 19,360 patients considered 30-day mortality in those receiving MBP+OAB versus OAB alone (Supplementary Figure 3, http://links.lww.com/SLA/B542), with the combination being associated with a significant reduction in 30-day mortality in all studies (RR 0.58, 95% CI 0.34–0.97, *P* = 0.04, I^2^ = 0%). However, no difference was observed in RCTs (RR 1.02, 95% CI 0.30–3.50, *P* = 0.97, I^2^ = 0%).

#### MBP+OAB Versus No Preparation

Just 2 cohort studies^31^,^46^ including 29,350 patients considered the impact of MBP+OAB versus no preparation on 30-day mortality. The combination of MBP+OAB was associated with a significant reduction in 30-day mortality (RR 0.36, 95% CI 0.17–0.76, *P* = 0.008, I^2^ = 46%).

#### Other Comparisons

Comparison of 30-day mortality between those receiving OAB versus no preparation and OAB versus MBP included just a single cohort study,^31^ thus meta-analysis was not conducted.

### Overall Morbidity

Only studies comparing MBP+OAB versus MBP alone were considered in terms of overall morbidity rates due to a paucity of data available for all other comparisons. When all 6 studies^31^,^61^,^62^,^66^,^68^,^76^ (32,568 patients) were compared, the combination of MBP+OAB was associated a significant reduction in overall morbidity (RR 0.67, 95% CI 0.63–0.71, *P* < 0.00001, I^2^ = 0%), as well as when evidence from cohort studies alone^31^,^68^ was considered (RR 0.67, 95% CI 0.63–0.71, *P* < 0.00001, I^2^ = 0%). However, with RCTs alone,^61^,^62^,^66^,^76^ there was no difference in overall morbidity between preparation methods (RR 0.71, 95% CI 0.41–1.24, *P* = 0.23, I^2^ = 9%).

### Development of Ileus

#### MBP+OAB Versus MBP

Five studies^31^,^43^,^51^,^53^,^54^ were included in the comparison of MBP+OAB versus MBP; 2 RCTs^51^,^53^ (879 patients) and 3 cohort studies (33,119 patients).^31^,^43^,^54^ Only 1 study^43^ provided a definition of ileus, with the other 4 studies^31^,^43^,^53^,^54^ not providing a definition. Overall, the combination of MBP+OAB was associated a significant reduction in the incidence of postoperative ileus (RR 0.72, 95% CI 0.52–0.98, *P* = 0.04, I^2^ = 36%). However, no difference was seen when just RCTs were considered (RR 0.62, 95% CI 0.14–2.67, *P* = 0.52, I^2^ = 50%) or cohort studies alone (RR 0.68, 95% CI 0.45–1.03, *P* = 0.07, I^2^ = 53%).

#### MBP+OAB Versus OAB

Three studies^31^,^71^,^83^ were included in the comparison between MBP+OAB versus OAB; 2 RCTs^71^,^83^ and 1 cohort study.^31^ None of these studies provided a definition for ileus. Overall, the combination of MBP+OAB was associated with a significant reduction in the incidence of postoperative ileus (RR 0.83, 95% CI 0.73–0.95, *P* = 0.008, I^2^ = 0%), mostly determined by the large single cohort study.^31^ However, no difference was seen when RCTs were considered (RR 1.25, 95% CI 0.68–2.33, *P* = 0.47, I^2^ = 0%).

#### MBP+OAB Versus No Preparation

No RCTs considered the comparison between MBP+OAB versus no preparation, with evidence arising from 2 cohort studies only.^31^,^41^ Only 1 study^41^ provided a definition of ileus. This demonstrated that the combination of MBP+OAB was associated with a significant reduction in ileus (RR 0.72, 95% CI 0.68–0.77, *P* < 0.00001, I^2^ = 0%).

#### Other Comparisons

The comparison in reoperation rates between OAB alone versus no preparation and OAB versus MBP were each only considered by 1 cohort study,^31^ thus meta-analysis was not performed.

### Reoperation

Insufficient data were available for any of the planned analyses on reoperation rates, with 2 studies including data comparing MBP+OAB versus MBP (1 RCT^49^ and 1 cohort study^31^), and just 2 studies comparing MBP+OAB versus OAB alone (again 1 RCT^71^ and 1 cohort study).^31^ Thus, no meta-analysis was performed. The comparisons of reoperation rates between MBP+OAB versus no preparation, OAB alone versus no preparation and OAB versus MBP were each only considered by 1 cohort study,^31^ and as such meta-analysis was not performed. However, the largest cohort study^31^ showed a significant reduction (*P* < 0.001) in reoperation rates with combined MBP+OAB (3.2%) compared with OAB alone (4.7%), MBP alone (4.2%), and no preparation (4.5%).

### *Clostridium difficile* Infection

#### MBP+OAB Versus MBP

Data on *Clostridium difficile* infection were sufficient only for the comparison between MBP+OAB versus MBP alone, with data from 14 studies, including 10 RCTs^53^,^55^,^61^,^62^,^65^,^67^,^69^,^75^,^78^,^80^ and 4 cohort studies.^43^,^54^,^68^,^82^ No difference in *C difficile* infection rates were seen when all evidence was considered (RR 0.94, 95% CI 0.55–1.61, *P* = 0.81, I^2^ = 37%), nor when just RCT studies or cohort studies alone were analyzed (RR 0.79, 95% CI 0.21–2.96, *P* = 0.72, I^2^ = 10% and RR 0.97, 95% CI 0.54–1.75, *P* = 0.92, I^2^ = 64%, respectively).

### Laparoscopic Versus Open Procedures

Nineteen RCTs^50^,^52^,^57^,^58^,^61^–^67^,^69^,^70^,^72^–^74^,^76^,^79^,^80^ provided data on SSI rates in patients undergoing open elective colorectal procedures between patients receiving combined MBP+OAB versus MBP alone, and 2 RCTs^53^,^55^ provided data on laparoscopic procedures alone. The remaining studies included either both open and laparoscopic procedures which could not be separated for analysis or did not state the surgical approach. No other comparison between preparations was considered due to a paucity of data. The combination of MBP+OAB versus MBP alone was associated with a significant reduction in SSI rates in patients undergoing an open resection (RR 0.55, 95% CI 0.44–0.69, *P* < 0.00001, I^2^ = 5%); however, no significant difference was seen in patients undergoing a laparoscopic procedure (RR 0.74, 95% CI 0.43–1.29, *P* = 0.29, I^2^ = 50%), although it should be borne in mind that this evidence was based upon 2 studies (1090 patients).

When anastomotic leak rates were compared between MBP+OAB versus MBP alone, divided by open and laparoscopic procedures, data could be analyzed from 9 RCTs^50^,^52^,^58^,^61^,^64^,^66^,^69^,^70^,^76^ in the open group and 2 RCTs^53^,^55^ in the laparoscopic group. There was no significant difference in anastomotic leak rates in either the open or laparoscopic groups (RR 0.69, 95% CI 0.30–1.60, *P* = 0.39, I^2^ = 13% and RR 0.68, 95% CI 0.28–1.65, *P* = 0.39, I^2^ = 0%, respectively).

## Discussion

### Main Findings

This meta-analysis has provided evidence to suggest that MBP+OAB should be given serious consideration in patients undergoing elective colorectal surgery to reduce the risk of SSI. In addition, it has shown that the combination of MBP+OAB is associated with significant reductions in anastomotic leak rates, 30-day mortality, overall morbidity, and the incidence of postoperative ileus, without increasing the risk of developing *C difficile* infection (Table 3). Its findings are in contradiction with previous meta-analyses^1^,^2^ that did not account for the role of luminal antibiotics and showed that MBP on its own was of no benefit when compared with no bowel preparation or rectal enemas alone.

However, as only 9.3% (6437 patients) of the 69,517 patients included were studied in the context of RCTs, the results must be interpreted with some caution. Hence, when evidence arising from RCTs alone was considered, the combination of MBP+OAB was associated with a significant reduction in SSI alone. The evidence for the combination of MBP+OAB to reduce SSI rates is, thus, strong. European data reporting the results of colorectal surgery in the context of Enhanced Recovery After Surgery protocols where mechanical bowel preparation is not used routinely, have shown SSI rates of >10%,^84^,^85^ whereas the US NSQIP studies have shown that SSI rates are approximately 3% with a combination of MBP+OAB, 6% with MBP alone and 7% with no preparation.^31^

When the combination of MBP+OAB was compared with OAB alone, a significant reduction in 30-day mortality and incidence of postoperative ileus was seen, but no difference was seen between the 2 preparations in RCTs alone. There are no RCTs focusing on the combinations of MBP+OAB versus no preparation, OAB alone versus no preparation or OAB alone versus MBP alone. However, evidence from cohort studies suggests that the combination of MBP+OAB versus no preparation is associated with a significant reduction in SSI, anastomotic leak, 30-day mortality, and postoperative ileus. For OAB versus no preparation, the only significant reduction was in SSI rates, and for OAB versus MBP there was no significant difference in any of the clinical outcome measures. When a planned subgroup analysis of patients undergoing open versus laparoscopic surgery was undertaken, the combination of MBP+OAB versus MBP alone was associated with a significant reduction in SSI rates in patients undergoing open procedures, but not in those undergoing laparoscopic procedures.

### Strengths and Weaknesses

The main weakness of this meta-analysis is the inclusion of both RCTs and cohort studies. While this lowers the overall quality of evidence, the decision to include cohort studies and large database studies was made as a large proportion of the recent evidence supporting the potential role of OAB or combined MBP+OAB has arisen from such studies. However, every analysis was conducted separately using evidence from RCTand cohort studies alone, as well as a summative analysis, to provide a more robust interpretation of the data.

The role of parenteral antibiotic prophylaxis is considered a standard of care in current practice, with evidence published in 1981^27^ providing evidence for its benefit in terms of infection prevention and overall mortality and dictating that no further placebo or no intervention trials should be conducted. Definitive support was provided in a Cochrane Review^86^ demonstrating a significant reduction in SSI in patients receiving parenteral antibiotic prophylaxis versus those receiving no antibiotics or placebo (RR 0.34, 95% CI 0.28–0.41, *P* < 0.0001).

The practice of mechanical bowel preparation has changed significantly since the early 1980s. The regimen of Lazorthes et al^62^ included admission 3 days prior to surgery and administration of a low-residue diet and standard mechanical procedures such as enemas and magnesium sulphate purges. In contrast, more modern regimens are typically administered the day before surgery and are less invasive. This is particularly important in the setting of prolonged starvation protocols in vogue prior to the more modern ones, as they resulted in increased preoperative dehydration and electrolyte disturbances which are known to have adverse effects on postoperative complications. It should, however, be considered that each study level comparison between preparation types should have been exposed to the same level of bias, thus making the results more comparable. The OAB agent, dosing, and timing as well as the parenteral antibiotic details were also inconsistent between studies, with insufficient data from each differing combination to perform a meaningful analysis. Several included just 1 preoperative dose of OAB, or differing parenteral antibiotic regimens depending upon which preparation regimen the patient received which exerts a potential significant bias. In addition, because of limited data, we have been unable to discern conclusively whether the reduction in morbidity is a result of OAB on their own or in combination with MBP.

The definition of anastomotic leak was not stipulated for inclusion within this meta-analysis, with the data from each individual study included, irrespective of whether this was based upon clinical or radiological diagnosis of anastomotic leak. However, the definition of leak was consistent within individual studies, thus the data from each study were comparable, attenuating this potential weakness.

### Interpretation of the Data in Context of Other Recent Studies

A recent meta-analysis^25^ included 23 RCTs and 8 cohort studies published between 1980 and 2015. However, multiple cohort studies arising from the NSQIP database were included within this study,^25^ and this probably represents multiple reporting of the same patient datasets. This study^25^ reported a significant reduction in SSI rates in patients included within cohort studies receiving MBP, OAB, and IV antibiotics versus those receiving MBP and IV antibiotics alone (RR 0.48, 95% CI 0.44–0.52, *P* = 0.00001, I^2^ = 45%). However, 4 of the 5 studies included within this analysis arose from the ACS NSQIP database. Bellows et al^23^ previously performed a meta-analysis on the role of oral nonabsorbable and intravenous antibiotics versus intravenous antibiotics alone in colorectal surgery, focusing on SSI. This study included 16 RCTs encompassing 2669 patients published between 1980 and 2011, with all studies including MBP within the protocol. This meta-analysis found that the combination of oral and IV antibiotics versus IV antibiotics alone was associated with a significant reduction in wound infection rates (RR 0.57, 95% CI 0.43–0.76, *P* = 0.0002, I^2^ = 19%), but no significant difference in anastomotic leak rates (RR 0.63, 95% CI 0.28–1.41, *P* = 0.3, I^2^ = 0%). The findings of the currently reported meta-analysis coincide with the results of these previous meta-analyses.

## Conclusion

The present meta-analysis is the largest and most comprehensive to date examining the role of bowel preparation prior to colorectal surgery, and supports a potentially significant benefit for OAB preparation, either in combination with MBP or alone, in the prevention of postoperative complications. While evidence arising from large retrospective cohort and database studies suggests a strong positive benefit, these are tempered when evidence arising from RCTs alone is considered. However, the evidence presented would suggest a benefit from OAB preparation in terms of SSI, which represents a major source of morbidity and increased healthcare costs. Further high-quality evidence is required to differentiate between the benefits of combined MBP+OAB or OAB alone in this setting before more definitive recommendations can be made.

## Supplementary Material

Supplemental digital content is available for this article. Direct URL citations appear in the printed text and are provided in the HTML and PDF versions of this article on the journal’s Web site (www.annalsofsurgery.com).

Supplemental Data File

## Figures and Tables

**Table 1:** Risk of bias within randomized controlled trials included within the meta-analysis

Reference	Random sequence generation	Allocation concealment	Blinding of participants and personnel	Blinding of outcome assessment	Incomplete outcome data	Selective reporting	Other bias
Anjum *et al*. 2017^49^	+	+	?	+	+	+	
Coppa *et al*. 1998^50^	?	?	-	+	-	-	
Espin-Basany *et al*. 2005^51^	?	?	?	+	+	+	
Hanel *et al*. 1980^52^	-	?	?	+	-	+	
Hata *et al*. 2016^53^	+	+	-	-	+	+	36 patients in the MBP+OAB group received reduced doses of kanamycin due to prescription error
Ikeda *et al*. 2016^55^	+	+	-	+	+	+	
Ishida *et al*. 2001^56^	+	-	-	-	+	?	
Kaiser *et al*. 1983^57^	?	+	+	+	+	-	Different IV antibiotic regimens given to the two groups
Khubchandani *et al*. 1989^58^	?	?	+	+	-	-	Different IV antibiotic regimens given to the two groups
Kobayashi *et al*. 2007^59^	+	?	-	-	-	-	
Lau *et al*. 1988^61^	+	?	?	?	+	+	
Lazorthes *et al*. 1982^62^	?	?	?	?	?	-	Different IV antibiotic regimens given to the two groups
Lewis 2002^63^	-	-	+	+	+	+	
McArdle *et al*. 1995^64^	?	?	?	?	+	?	Different IV antibiotic regimens given to the two groups
Monrozies *et al*. 1983^65^	?	?	?	?	+	+	Different IV antibiotic regimens given to the two groups
Nohr *et al*. 1990^66^	?	?	+	+	-	+	Different IV antibiotic regimens given to the two groups
Oshima *et al*. 2013^67^	?	?	-	-	+	+	
Peruzzo *et al*. 1987^69^	?	?	?	?	+	+	
Playforth *et al*. 1988^70^	?	?	?	?	+	+	
Ram *et al*. 2005^71^	-	?	?	?	+	+	
Reddy *et al*. 2007^72^	+	+	-	-	+	+	Group also randomized to probiotics – not included within meta-analysis
Reynolds *et al*. 1989^73^	+	-	?	?	-	-	Two different IV antibiotic regimens in the MBP group
Sadahiro *et al*. 2014^75^	+	-	+	+	?	?	Group also randomized to probiotics – not included within meta-analysis
Stellato *et al*. 1990^76^	+	?	+	+	-	+	
Takesue *et al*. 2000^78^	?	?	?	?	-	?	
Taylor *et al*. 1994^79^	?	?	-	-	-	+	
Uchino *et al*. 2017^80^	+	+	-	+	-	?	*C. difficile* toxin and faecal cultures only pre-op
Zmora *et al*. 2003^83^	+	+	?	?	-	+	

+ Low risk of bias; - High risk of bias; ? Unclear risk of bias

**Table 2:** Patient demographics in studies included

Reference	Study methodology	Number of patients	Indication for surgery	Location of resection	Laparoscopic or open	OAB agent	MBP agent	Parenteral agent	Comparison included
Anjum *et al.* 2017^49^	RCT	190	Gastrointestinal tract fistula IBD Trauma Malignancy	Partial small bowel resection – 39 Right colectomy – 67 Left colectomy – 50 LAR – 34	Laparoscopic – 40 Open - 150	Metronidazole 400 mg and levofloxacin 200 mg TDS on the day before surgery.	Sodium phosphate 133 ml twice a day on the day before surgery.	Second generation cephalosporin + metronidazole 30-60 min pre-incision, every 3 h intra-op then 24 h post-op.	MBP+OAB vs. MBP
Cannon *et al.* 2012^45^	Retrospective database study – Veterans Affairs Surgical Quality Improvement Program	9940	Neoplasm – 7871 IBD – 176 Diverticulitis – 644 Not stated – 1248	Ileocolic resection – 984 Partial colectomy – 6847 Rectal resection – 1771 Total colectomy – 338	Not stated	Erythromycin, neomycin or metronidazole.	Polyethylene glycol, phospho-soda or magnesium citrate.	Not stated	MBP+OAB vs. MBP MBP+OAB vs. OAB MBP+OAB vs. no prep OAB vs. no prep OAB vs. MBP
Coppa *et al.* 1988^50^	RCT	350	Cancer – 255 Inflammatory – 46 Other – 9	Not stated	All open	Neomycin 8 g/day and erythromycin 4 g/day in divided doses for 24 h pre-op.	Fleet phospho-soda between 1 and 3 days pre-op, and saline enemas for the last two days.	Cefoxitin 1-2 g according to patient body weight given preoperatively, intraoperatively and every 6 h for the first post-op day.	MBP+OAB vs. MBP
Englesbe *et al.* 2010^43^	Retrospective propensity-matched database study – Michigan Surgical Quality Collaborative – Colectomy Best Practices Project	740	Not stated	Segmental colectomy Ileocolic resection	Open and laparoscopic	Neomycin and erythromycin 76.3% Neomycin alone 7.9% Erythromycin alone 2.6% Metronidazole alone 2.6% Clindamycin alone 2.6%	Polyethylene glycol 20.9% Phospho-soda 5.9% Fleet enema 38.5% Magnesium citrate 5% Other 29.7%	Not stated	MBP+OAB vs. MBP
Espin-Basany *et al.* 2005^51^	RCT	300	Cancer – 269 IBD – 4 Diverticular disease – 21 Not stated – 6	Segmental resection – 120 Sigmoidectomy – 69 Anterior resection – 27 TME-coloanal – 66 APR – 18	Not stated	Neomycin 1 g and metronidazole 1 g EITHER TDS the day before surgery OR OD the day before surgery.	Sodium phosphate 45 ml diluted in 90 ml water BD the day before surgery	Cefoxitin 1 g pre-incision and two doses at 8 and 16 h post-op.	MBP+OAB vs. MBP
Hanel *et al.* 1980^52^	RCT	77	Adenoma – 2 Carcinoma – 48 IBD – 4 Diverticular disease – 7 Hodgkin’s disease – 1 Villus papilloma – 1 Cecal volvulus – 2 Sigmoid volvulus – 2	Right colectomy – 15 Left colectomy – 6 Transverse colectomy – 2 Sigmoid colectomy – 10 Colonic bypass – 1 Cecostomy – 1 Colostomy – 1 Colostomy closure – 5 Colotomy and polypectomy – 2 Anterior resection – 14 APR – 7 Proctocolectomy – 2	All open	Metronidazole 1 g QDS for four days and neomycin 1 g TDS for two days prior to surgery.	Four day standard mechanical preparation including a low residue diet, and alternating enemas or washouts.	Clindamyin 7 mg/kg and cephazolin sodium 1 g given at the start of the anesthetic.	MBP+OAB vs. MBP
Hata *et al.* 2016^53^	RCT	579	Colorectal malignancy Adenoma	Colectomy – 376 Anterior resection – 183 APR – 20	All laparoscopic	Kanamycin 1g and metronidazole 750 mg BD at 13 h and 9 h pre-op.	Sodium picosulphate 75 mg and magnesium citrate 34 g with 180 ml water the day before surgery.	Cefmetazole 1 g 30 min pre-incision then every 3 h intra-op.	MBP+OAB vs. MBP
Ichimanda *et al.* 2017^54^	Retrospective case controlled series	344	All colorectal cancer	Not stated. Primary site: Colon – 181 Rectum – 163	Laparoscopic – 293 Open - 51	Kanamycin 1 g TDS and metronidazole 1 g TDS for 24 h prior to surgery.	Polyethylene glycol 2 L and nasoside (Pulsenide) 24 mg.	Second generation cephem on the day of surgery until the second post-op day.	MBP+OAB vs. MBP
Ikeda *et al.* 2016^55^	RCT	511	Colorectal malignancy	Colonic surgery – 309 Anterior resection – 177 APR - 25	All laparoscopic	Kanamycin 1 g and metronidazole 750 mg BD the day before surgery.	Magnesium citrate and sodium picosulphate the day before surgery.	Cefmetazole 1 g at least 30 min pre-incision, every 3 h intra-op and for 24 h post-op.	MBP+OAB vs. MBP
Ishida *et al.* 2001^56^	RCT	143	Cancer – 135 IBD – 4 Diverticular disease – 1 Not stated – 3	Colectomy – 76 Anterior resection – 47 APR – 9 Total proctectomy with J pouch – 3 Total pelvic exenteration – 4 Other – 4	Not stated	Kanamycin 2 g/day and erythromycin 1.6 g/day in 4 divided doses from 2 days prior to surgery.	Polyethylene glycol 2 L given the day before surgery.	Cefotiam 1 g after induction, 1 g at one hour after completion of surgery and 4 additional doses given BD for 2 consecutive days.	MBP+OAB vs. MBP
Kaiser *et al.* 1983^57^	RCT	119	Local malignancy – 50 Metastatic malignancy – 30 Diverticulitis - 17 Polyps – 9 IBD – 9 Not stated – 4	Right colectomy – 34 Left colectomy – 25 Sigmoid resection – 25 APR – 11 Anterior resection – 7 Subtotal colectomy – 6 Operative colotomy – 6 Total colectomy – 3 Colostomy closure - 2	All open	Neomycin 1 g TDS and erythromycin 1 g TDS the day prior to surgery.	Magnesium citrate and cleansing enemas for 2 days prior to surgery.	Cefoxitin 2 g with the ‘on call’ medications, 1 g intra-operatively and 1 g every 6 h following surgery for four doses in the MBP alone group. Cefazolin 1 g with the ‘on call’ medications, 500 mg intra-operatively and 1 g every 6 h following surgery for four doses in the MBP+OAB group.	MBP+OAB vs. MBP
Khubchandani *et al.* 1989^58^	RCT	155	‘Colonic surgery’	Not stated	All open	Neomycin 1 g and erythromycin 1 g at 1 pm, 2 pm and 10 pm the day before surgery.	Castor oil 60 ml the afternoon of admission and saline enemas the night of admission and the following morning until the effluent was clear.	Metronidazole 1 g given 1 h before surgery, then 500 mg at 6 and 12 h post-op in MBP alone group. Cefazolin 1 g given 1 h before surgery, then 1 g at 6 and 12 h post-op in the MBP+OAB group.	MBP+OAB vs. MBP
Kim *et al.* 2014^41^	Retrospective propensity-matched database study – Michigan Surgical Quality Collaborative – Colectomy Best Practices Project	1914	Not stated	Ileocolic resection with anastomosis Segmental colectomy with anastomosis	Open – 1049 Laparoscopic – 865	Not stated	Not stated	Not stated	MBP+OAB vs. no prep
Kobayashi *et al.* 2007^59^	RCT	484	Colorectal malignancy	Surgical procedure: Colon – 241 Rectum – 243	Not stated	Kanamycin 1 g and erythromycin 400 mg TDS the day before surgery.	Polyethylene glycol 2 L the morning of the day before surgery.	Cefmetazole 1 g at induction, an additional dose if operation exceeded 3 h, then BD for 3 days post-op.	MBP+OAB vs. MBP
Konishi *et al.* 2006^60^	Retrospective case controlled series – National Nosocomial Infection Surveillance program	556	Not stated	Right colectomy – 94 Left colectomy – 155 Other colectomy – 90 LAR – 126 APR – 51 Total colectomy or panproctocolectomy – 34 Hartmann’s procedure – 6 Additional concomitant procedures: Ostomy closure – 47 Ostomy formation – 106 Multiple organ resection – 93	Open - 515 Laparoscopic – 41	Kanamycin and metronidazole.	Oral laxative and glycerine enema.	Second generation cephalosporin given 30 min prior to incision, repeated every 3 h intra-op and stopped within 24 h after the operation.	MBP+OAB vs. MBP
Lau *et al.* 1988^61^	RCT	194	All cancer	Right colectomy – 39 Left colectomy – 7 Transverse colectomy – 9 Sigmoid colectomy – 22 Subtotal colectomy – 10 Pelvic exenteration – 2 Palliative bypass – 4 Anterior resection – 39 LAR – 17 APR – 45	All open	Neomycin 1 g and erythromycin 1 g at 1 pm, 2 pm and 11 pm the day prior to surgery.	3 days of oral bisacodyl, magnesium sulphate and saline enemas prior to surgery.	Metronidazole 500 mg and gentamycin 2mg/kg body weight given 30 min prior to surgery, then repeated at 8 h intervals for two further doses.	MBP+OAB vs. MBP
Lazorthes *et al.* 1982^62^	RCT	90	Cancer – 51 Colostomy closure – 23 Benign disease – 16	Colectomy – 30 APR – 9 Sphincter-saving resection – 23 Miscellaneous – 28	All open	Kanamycin 1 g QDS and metronidazole 250 mg QDS for 3 days prior to surgery.	Three days of low residue diet, enemas and magnesium sulphate purges.	Cephradine 2 g at induction with metronidazole 500 mg infusion over 4 h in MBP alone group. Cephradine 2 g and gentamycin 2 mg/kg as IM injection at time of premedication in the MBP+OAB group.	MBP+OAB vs. MBP
Lewis 2002^63^	RCT	208	Cancer – 150 IBD – 51 Rectal prolapse – 10 Not stated – 2 (5 patients withdrawn)	Anterior resection – 119 APR – 19 Right colectomy – 55 Left colectomy – 13 Transverse colectomy – 4	Not stated	Neomycin 2 g and metronidazole 2 g BD the day before surgery.	Sodium phosphate the day before surgery, with saline enemas if this did not result in a clear effluent.	Amikacin 1 g and metronidazole 1 g on the day of surgery.	MBP+OAB vs. MBP
McArdle *et al.* 1995^64^	RCT	169	Cancer/cancer related – 151 IBD – 13 Diverticular disease – 5	Right colectomy – 35 Left colectomy – 26 Anterior resection – 24 APR – 17 Total colectomy – 5 Hartmann’s procedure/reversal – 15 Bypass – 7 Small bowel resection – 14 Formation or revision of stoma – 18 Others – 8	All open	Ciprofloxacin 1 g 1 h prior to surgery – one group received no further doses and one group received ciprofloxacin 750 mg BD for 3 days.		MBP alone: Gentamycin 120 mg + metronidazole 500 mg at induction then one group received gentamycin 80 mg + metronidazole 500 mg at 8 and 16 h post-op and one group received gentamycin 80 mg + metronidazole 500 mg TDS for 3 days. MBP+OAB: metronidazole 500 mg at induction then in one group at 8 and 16 h post-op and in the other metronidazole 500 mg TDS for 3 days.	MBP+OAB vs. MBP
Midura *et al.* 2018^31^	Database study – ACS NSQIP	45,724	IBD Cancer Diverticulitis Others	Left colectomy Right colectomy Segmental colectomy	Open Laparoscopic Robotic	Not stated	Not stated	Not stated	MBP+OAB vs. MBP MBP+OAB vs. OAB MBP+OAB vs. no prep MBP vs. OAB OAB vs. no prep
Mik *et al.* 2016^46^	Retrospective cohort study	2240	Colorectal malignancy	Right colectomy – 413 Left colectomy – 171 Sigmoidectomy – 282 Hartmann’s – 171 Anterior resection – 309 LAR – 381 APR – 163 Not stated – 350	All open	Erythromycin 500 mg and neomycin 500 mg TDS the day before surgery.	Oral macrogol the day before surgery.	Cefazolin 1 g and metronidazole 500 mg directly before incision, and broadened to 3 doses if surgery lasted longer than 3 h.	MBP+OAB vs. no prep
Monrozies *et al.* 1983^65^	RCT	60	Cancer – 34 Closure of colostomy – 8 Benign – 18	Colectomy – 35 Rectal surgery – 15 Others – 10	All open	Kanamycin 1 g QDS and metronidazole 1 g QDS for 3 days pre-op.	Magnesium sulphate and enemas.	MBP+OAB: Cephradine 2 g at induction and IM gentamycin 2 mg/kg at premedication according to patient body weight. MBP alone: cephradine 2 g at induction and 500mg metronidazole infusion then two further infusions within 24 h of metronidazole.
Nohr *et al.* 1990^66^	RCT	149	Cancer – 116 Complicated diverticulitis – 9 Crohn’s disease – 8 UC – 1 Not stated – 15	Right colectomy – 29 Rectal resection – 44 Sigmoid resection – 30 APR – 19 Others – 27	All open	Bacitracin 250 mg and neomycin 250 mg TDS for 2 days pre-op. Metronidazole 500 mg TDS the day before surgery.	Frangula bark 2 tablets 2 days pre-op and magnesium sulphate (7.5 g) daily for 2 days pre-op.	Ampicillin 1 g within 1 h pre-op in MBP+OAB group. Fosfomycin 8 g and metronidazole 1 g within 1 h pre-op in MBP alone group.	MBP+OAB vs MBP
Oshima *et al.* 2013^67^	RCT	200	Ulcerative colitis	Restorative proctocolectomy with ileal pouch-anal anastomosis (IPAA)	All open	Kanamycin 500 mg and metronidazole 500 mg TDS the day before surgery.	Magnesium citrate 1.8 L the day before surgery.	Flomoxef 30 min before surgery, repeated every 3 h intra-op and then 24 h post-op.	MBP+OAB vs. MBP
Ozdemir *et al.* 2016^68^	Retrospective cohort study	90	Colonic malignancy Ulcerative colitis	Right colectomy – 17 Left colectomy – 10 Transverse colectomy – 9 LAR – 45 Total colectomy – 8 Other – 1	All open	Gentamycin 240 ml and metronidazole 2 g at 11 and 9 h pre-op.	Sodium dibasic phosphate 45 ml BD at 12 and 10 h pre-op, fleet enema 8 and 3-4 h pre-op.	Cefazolin 1 g and metronidazole 500 mg during anesthetic induction, continued BD for 5 days post-op.	MBP+OAB vs. MBP
Peruzzo *et al.* 1987^69^	RCT	80	Cancer – 61 Diverticular disease – 6 Colostomy – 12 Not stated – 1	Right colectomy – 17 Left colectomy – 27 Sigmoid colectomy – 9 Anterior resection – 13 APR – 2 Colostomy closure - 12	All open	Neomycin 1 g at 19, 18 and 9 h pre-op and 2 g oral tinidazole.	‘According to standard practice’.	Cefoxitin 30 min pre-op then at 6 and 12 h post-op.	MBP+OAB vs. MBP
Playforth *et al.* 1988^70^	RCT	119 + 83 non randomized cohort (not included)	Cancer (curative) - 66 Cancer (palliative) - 22 Inflammatory - 31	Right colon – 38 Left colon and rectum - 81	All open	Neomycin 1 g every 6 h and metronidazole 200 mg every 8 h for 24 h prior to surgery.	Mannitol 100 g in 1 L water the day before surgery.	Metronidazole 500 mg at the time of premedication.	MBP+OAB vs. MBP
Ram *et al.* 2005^71^	RCT	329	Cancer – 268 Benign - 61	Right colectomy – 42 Left colectomy – 74 Sigmoidectomy – 86 Subtotal colectomy – 11 APR – 34 Transverse colectomy – 3 Anterior resection – 50 LAR – 29	All open	Not stated	Monobasic sodium phosphate 2.4 g and dibasic sodium phosphate 0.9 g given the day before surgery.	Metronidazole 500 mg and ceftriaxone 1 g given 1 h pre-induction and continued for 48 h post-op.	MBP+OAB vs. OAB
Reddy *et al.* 2007^72^	RCT	92 (46 pertinent to this meta-analysis)	Cancer and benign	Right colectomy- 16 Left colectomy - 6 Anterior resection - 18 APR - 3 Subtotal colectomy - 2 Panproctocolectomy - 1	All open	3 g neomycin in three divided doses the day before surgery.	Sodium picosulphate and magnesium citrate given the day before surgery.	Not stated	MBP+OAB vs. MBP
Reynolds *et al.* 1989^73^	RCT	330	Cancer – 247 Benign – 5 Inflammatory lesion – 19 Others – 59	Right colectomy – 65 Left colectomy – 9 Sigmoid colectomy – 48 APR – 50 Anterior resection – 97 Panproctocolectomy – 2 Subtotal colectomy – 9 Hartmann’s procedure – 10 Colostomy surgery – 35 Other – 5	All open	Metronidazole 400 mg eight hourly and neomycin 1 g six hourly for 48 h prior to surgery. Last dose of antibiotics given 8 and 12 h prior to surgery, respectively.	Magnesium sulphate up to 8×4 g doses for 48 h starting 72 h pre-op. Followed by two doses of sodium picosulphate the day before surgery.	Either piperacillin 2 g IV at induction and 3 further doses 8 hourly or metronidazole 500 mg and cefuroxime 1.5 g at induction followed by 3 further doses of metronidazole and 2 further doses of cefuroxime.	MBP+OAB vs. MBP
Rohwedder *et al.* 1993^74^	Retrospective historical case controlled series	818 (100 MBP+OAB, 718 MBP)	Of those with MBP+OAB: Colorectal cancer – 89 Anal cancer – 1 Pelvic recurrence – 1 Villous tumour – 1 Diverticular disease – 6 UC – 1 Crohn’s colitis – 1	Of those with MBP+OAB: Right colectomy – 14 Left colectomy – 25 LAR – 37 Miles APR – 12 Total colectomy – 6 Subtotal colectomy – 1 Double colectomy – 2 Other – 3	All open	Ciprofloxacin 750 mg taken between 1 and 3 h pre-op.	Polyethylene glycol the day before surgery.	Gentamycin 80 mg and metronidazole 500 mg at the beginning of induction, then gentamycin 80 mg every 8 h for 3 days.	MBP+OAB vs. MBP
Sadahiro *et al.* 2014^75^	RCT	294	Colorectal malignancy	Not stated – tumour location: Right colon – 99 Transverse colon – 38 Left colon – 157	Open – 214 Laparoscopic – 80	Kanamycin sulphate 500 mg + metronidazole 500 mg TDS the day before surgery.	Sodium picosulphate 10 ml 2 days pre-op and 2 L polyethylene glycol the day before surgery.	Flomoxef 1 g 1 h pre-incision and further dose given if operative duration exceeded 3 h.	MBP+OAB vs. MBP
Stellato *et al.* 1990^76^	RCT	146	Cancer – 123 Polyp – 11 Diverticular disease – 6 IBD – 6	Right colectomy - 44 Left colectomy – 17 Transverse colectomy - 4 Sigmoid colectomy - 30 LAR - 31 APR - 15 Subtotal colectomy - 5	All open	Neomycin 1g and erythromycin 1g TDS on the day before surgery.	Magnesium citrate 1.745 g in 296 ml in the morning and an enema (19 g sodium biphosphate and 7 g sodium phosphate in 118 ml) in the evening 2 days prior to surgery. Magnesium citrate 1.745 g in 296 ml in the morning and saline enemas until clear in the evening of the day before surgery.	Cefoxitin 2 g at induction then at 6 and 12 h following the first dose.	MBP+OAB vs. MBP
Sun *et al.* 2018^77^	Retrospective case controlled series	321	Malignancy – 306 Benign – 12 IBD – 3	Right colectomy – 86 Left colectomy – 24 Sigmoid colectomy – 65 LAR – 90 APR – 16 Laparoscopic anterior resection – 12 Laparoscopic sigmoidectomy – 15 Subtotal colectomy – 4 Laparoscopic right hemicolectomy – 8	Laparoscopic - 35 Open - 269	Neomycin 1 g and erythromycin 1 g at 20, 19 and 10 h prior to surgery.	Fleet phospho-soda 45 ml at 24 and 15 h before surgery then tap water enema at 2 h pre-op.	Cefazolin 1 g at induction.	MBP+OAB vs. MBP
Takesue *et al.* 2000^78^	RCT	83	Dukes A – 16 Dukes B – 43 Dukes C – 24	Ileocecal resection – 5 Right colectomy – 14 Left colectomy – 3 Transverse colectomy – 6 Sigmoidectomy – 24 LAR – 24 Miles’ APR – 7	All open	Kanamycin 500 mg and metronidazole 500 mg at 2 pm, 3 pm and 11 pm the day before surgery.	Polyethylene glycol commence at 10 am the day before surgery.	Cefmetazole 1 g given at induction, then TDS for 3 days following surgery.	MBP+OAB vs. MBP
Taylor *et al.* 1994^79^	RCT	327	Benign – 53 Cancer – 259 IBD – 15	Anastomosis right colon – 93 Anastomosis left colon/rectum – 168 Hartmann’s resection – 6 APR – 43 Not stated – 17	Not stated	Ciprofloxacin 500 mg BD the day before surgery.	Sodium picosulphate one sachet BD the day before surgery.	Piperacillin 4 g at induction of anesthesia.	MBP+OAB vs. MBP
Uchino *et al.* 2017^80^	RCT	325	Crohn’s disease	Small bowel resection Colonic resection Rectal resection	All open	Kanamycin 500 mg and metronidazole 500 mg TDS the day before surgery.	Sodium picosulphate hydrate (20 ml of 0.75%) pre-operatively.	Flomoxef sodium 30 min before surgery, every 3 h intra-op then 24 h post-op.	MBP+OAB vs. MBP
Vo *et al.* 2018^81^	Retrospective case control series	89	Colorectal cancer	Left colectomy - 14 Sigmoid colectomy – 16 LAR – 35 APR – 14 Subtotal colectomy or other – 10	Open - 21 Minimally invasive – 68	Neomycin sulphate 1 g and metronidazole hydrochloride 1 g TDS. Commenced one day prior to surgery.	Magnesium citrate 296 ml twice daily. Commenced one day prior to surgery.	Ertapenem – 82 Non-ertapenem - 7	MBP+OAB vs. MBP
Wren *et al*. 2005^82^	Retrospective case controlled study	304	Not stated	Colon and/or rectal resection – 258 Colostomy creation or take down – 46	Open and laparoscopic	Neomycin 1 g and erythromycin 1 g	GoLYTELY, magnesium citrate or Fleet phospho-soda	Cephalosporin and metronidazole 59.2% Second generation cephalosporin 21.0% Fluoroquinolone and metronidazole or clindamycin 9.5% First-generation cephalosporin alone 3.9% Extended-spectrum penicillin 3.6%	MBP+OAB vs. MBP
Zmora *et al.* 2003^83^	RCT	380	Cancer – 296 Diverticular disease – 16 Hartmann’s procedure (for closure) – 29 Benign polyp – 14 IBD – 13 Not stated – 12	Right colectomy – 113 Left colectomy – 33 Sigmoidectomy – 89 Anterior resection – 83 Closure of Hartmann’s – 29 Subtotal/total abdominal colectomy – 24 Total proctectomy and ileal pouch – 9	Not stated	Neomycin and erythromycin	Polyethylene glycol 1 gallon 12 to 16 h pre-op. Rectal surgery – given Fleet enema.	‘Broad spectrum antibiotics’ continued for 24 h post-op.	MBP+OAB vs. OAB

APR – abdominoperineal resection; IBD – inflammatory bowel disease; LAR – low anterior resection; MBP – mechanical bowel preparation; OAB – oral antibiotics; RCT – randomized controlled trial

**Table 3:** Overall summary of results

Preparation considered	Outcome measure	All studies	RCTs only	Cohort studies only
MBP+OAB *vs*. MBP	Surgical site infection	Significant ↓ with MBP+OAB (RR 0.51, 95% CI 0.46 to 0.56, p<0.00001, I^2^=13%)	Significant ↓ with MBP+OAB (RR 0.57, 95% CI 0.48 to 0.68, p<0.00001, I^2^=12%)	Significant ↓ with MBP+OAB (RR 0.48, 95% CI 0.44 to 0.51, p<0.00001, I^2^=0%
	Anastomotic Leak	Significant ↓ with MBP+OAB (RR 0.62, 95% CI 0.55 to 0.70, p<0.00001, I^2^=0%)	No difference (RR 0.69, 95% CI 0.43 to 1.11, p=0.13, I^2^=0%)	Significant ↓ with MBP+OAB (RR 0.45, 95% CI 0.25 to 0.80, p=0.007, I^2^=22%)
	30-day mortality	Significant ↓ with MBP+OAB (RR 0.58, 95% CI 0.44 to 0.76, p<0.0001, I^2^=0%)	No difference (RR 0.66, 95% CI 0.35 to 1.25, p=0.20, I^2^=0%)	Significant ↓ with MBP+OAB (RR 0.56, 95% CI 0.42 to 0.76, p=0.0002, I^2^=0%)
	Overall morbidity	Significant ↓ with MBP+OAB (RR 0.67, 95% CI 0.63 to 0.71, p<0.00001, I^2^=0%)	No difference (RR 0.71, 95% CI 0.41 to 1.24, p=0.23, I^2^=9%)	Significant ↓ with MBP+OAB (RR 0.67, 95% CI 0.63 to 0.71, p<0.00001, I^2^=0%)
	Development of ileus	Significant ↓ with MBP+OAB (RR 0.72, 95% CI 0.52 to 0.98, p=0.04, I^2^=36%)	No difference (RR 0.62, 95% CI 0.14 to 2.67, p=0.52, I^2^=50%)	No difference (RR 0.68, 95% CI 0.45 to 1.03, p=0.07, I^2^=53%)
	*C. difficile* infection	No difference (RR 0.94, 95% CI 0.55 to 1.61, p=0.81, I^2^=37%)	No difference (RR 0.79, 95% CI 0.21 to 2.96, p=0.72, I^2^=10%)	No difference (RR 0.97, 95% CI 0.54 to 1.75, p=0.92, I^2^=64%)
MBP+OAB *vs*. OAB	Surgical site infection	No difference (RR 0.98, 95% CI 0.64 to 1.50, p= 0.92, I^2^=77%)	No difference (RR 1.36, 95% CI 0.78 to 2.35, p=0.28, I^2^=0%)	No difference (RR 0.83, 95% CI 0.48 to 1.43, p=0.51, I^2^=90%)
	Anastomotic Leak	No difference (RR 0.79, 95% CI 0.59 to 1.05, p=0.11, I^2^=0%)	No difference (RR 1.39, 95% CI 0.47 to 4.10, p=0.55, I^2^=0%)	---
	30-day mortality	Significant ↓ with MBP+OAB (RR 0.58, 95% CI 0.34 to 0.97, p=0.04, I^2^=0%)	No difference (RR 1.02, 95% CI 0.30 to 3.50, p=0.97, I^2^=0%)	---
	Overall morbidity	---	---	---
	Development of ileus	Significant ↓ with MBP+OAB (RR 0.83, 95% CI 0.73 to 0.95, p=0.008, I^2^=0%)	No difference (RR 1.25, 95% CI 0.68 to 2.33, p=0.47, I^2^=0%)	---
	*C. difficile* infection	---	---	---
MBP+OAB *vs*. no preparation	Surgical site infection	---	---	Significant ↓ with MBP+OAB (RR 0.54, 95% CI 0.43 to 0.68, p<0.00001, I^2^=82%)
	Anastomotic Leak	---	---	Significant ↓ with MBP+OAB (RR 0.52, 95% CI 0.45 to 0.59, p<0.00001, I^2^=0%)
	30-day mortality	---	---	Significant ↓ with MBP+OAB (RR 0.36, 95% CI 0.17 to 0.76, p=0.008, I^2^=46%)
	Overall morbidity	---	---	---
	Development of ileus	---	---	Significant ↓ with MBP+OAB (RR 0.72, 95% CI 0.68 to 0.77, p<0.00001, I^2^=0%)
	*C. difficile* infection	---	---	---

MBP – mechanical bowel preparation; OAB – oral antibiotics; --- Insufficient data for conduct of meta-analysis OAB *vs*. no preparation – only outcome was surgical site infection in cohort studies alone which demonstrated a significant ↓ with OAB. OAB *vs*. MBP – only outcome was surgical site infection in cohort studies alone which demonstrated no difference.

## Mini Abstract

This meta-analysis examines the role of oral antibiotic preparation, with and without mechanical bowel preparation in elective colorectal surgery. Combined oral antibiotic and mechanical bowel preparation significantly reduces surgical site infection rates *versus* mechanical preparation alone, but this effect is similar to that of oral antibiotics alone.
