# Supplementary material for: The Role of Oral Antibiotic Preparation in Elective Colorectal Surgery: A Meta-analysis
Source: Ann Surg. Author manuscript; Available in PMC 2020 Jul 1. (PMC6570620; doi:10.1097/SLA.0000000000003145)
Supplement: Supplemental Data File [file EMS81356-supplement-Supplemental_Data_File.docx]

**Supplementary Digital Material**

**Supplementary Fig 1** – PRISMA diagram demonstrating identification of studies from the initial literature search.


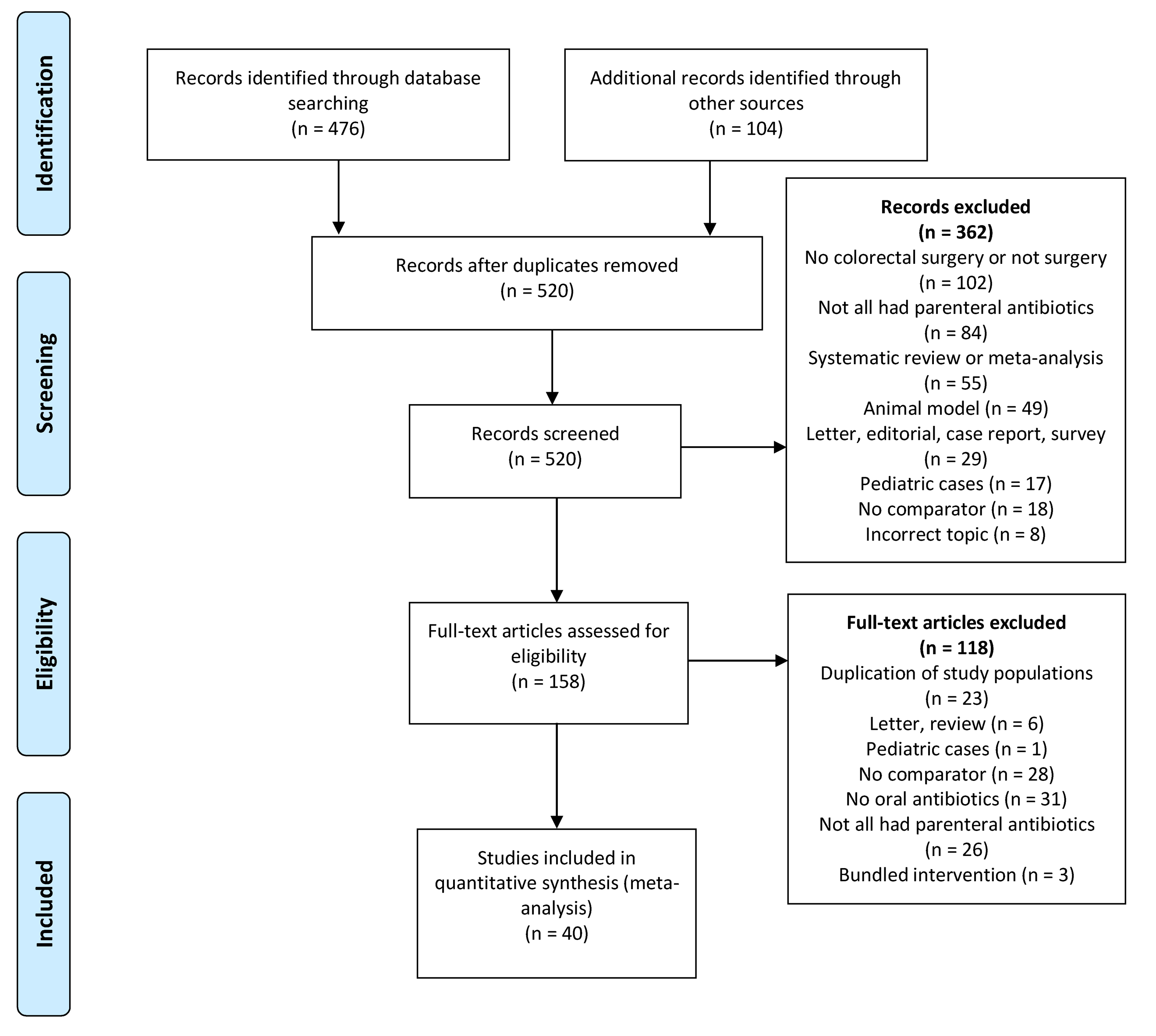


**Supplementary Fig 2** - Forest plot comparing anastomotic leak rate for patients receiving MBP+OAB *versus* OAB alone, divided by evidence from RCTs and cohort studies. A Mantel-Haenszel random effects model was used to perform the meta-analysis and risk ratios are quoted including 95% confidence intervals.


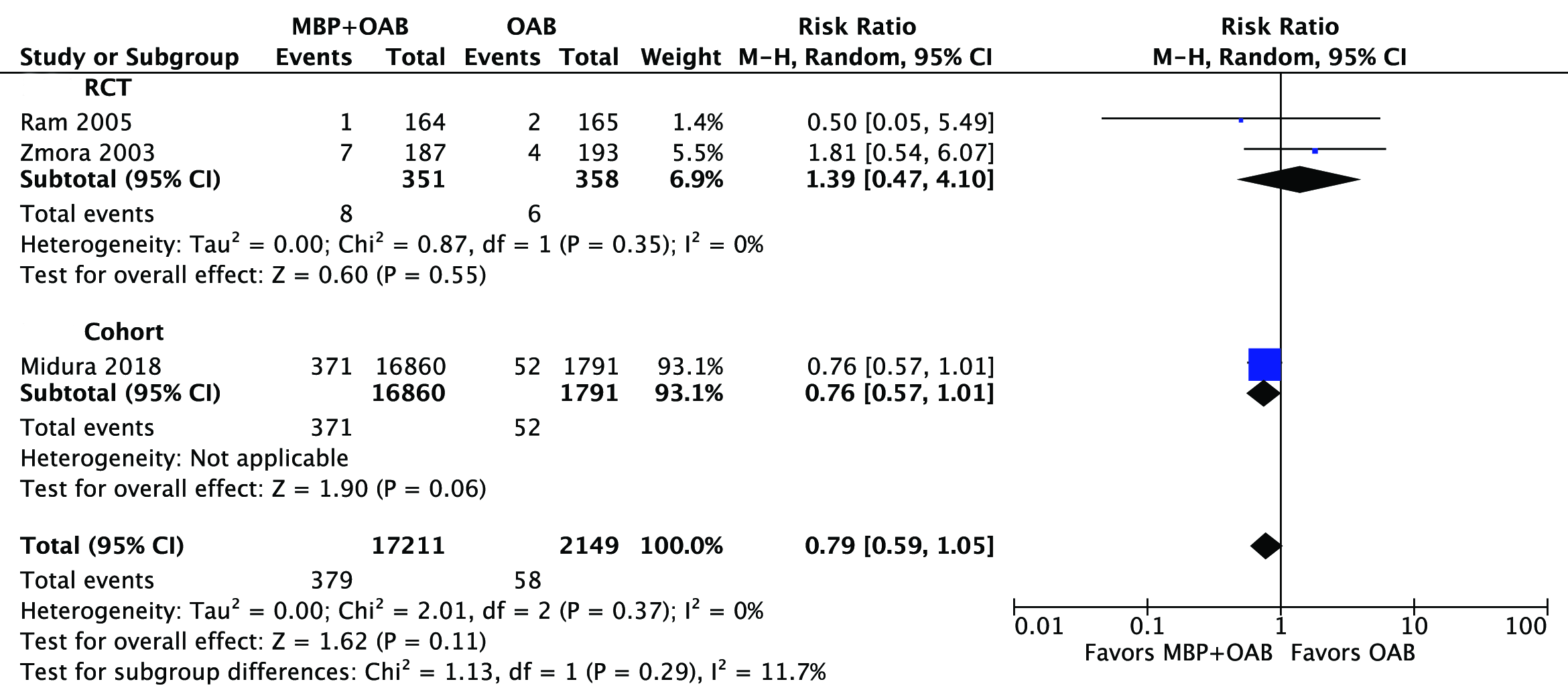


**Supplementary Fig 3** - Forest plot comparing 30-day mortality rates for patients receiving MBP+OAB *versus* OAB alone, divided by evidence from RCTs and cohort studies. A Mantel-Haenszel random effects model was used to perform the meta-analysis and risk ratios are quoted including 95% confidence intervals.


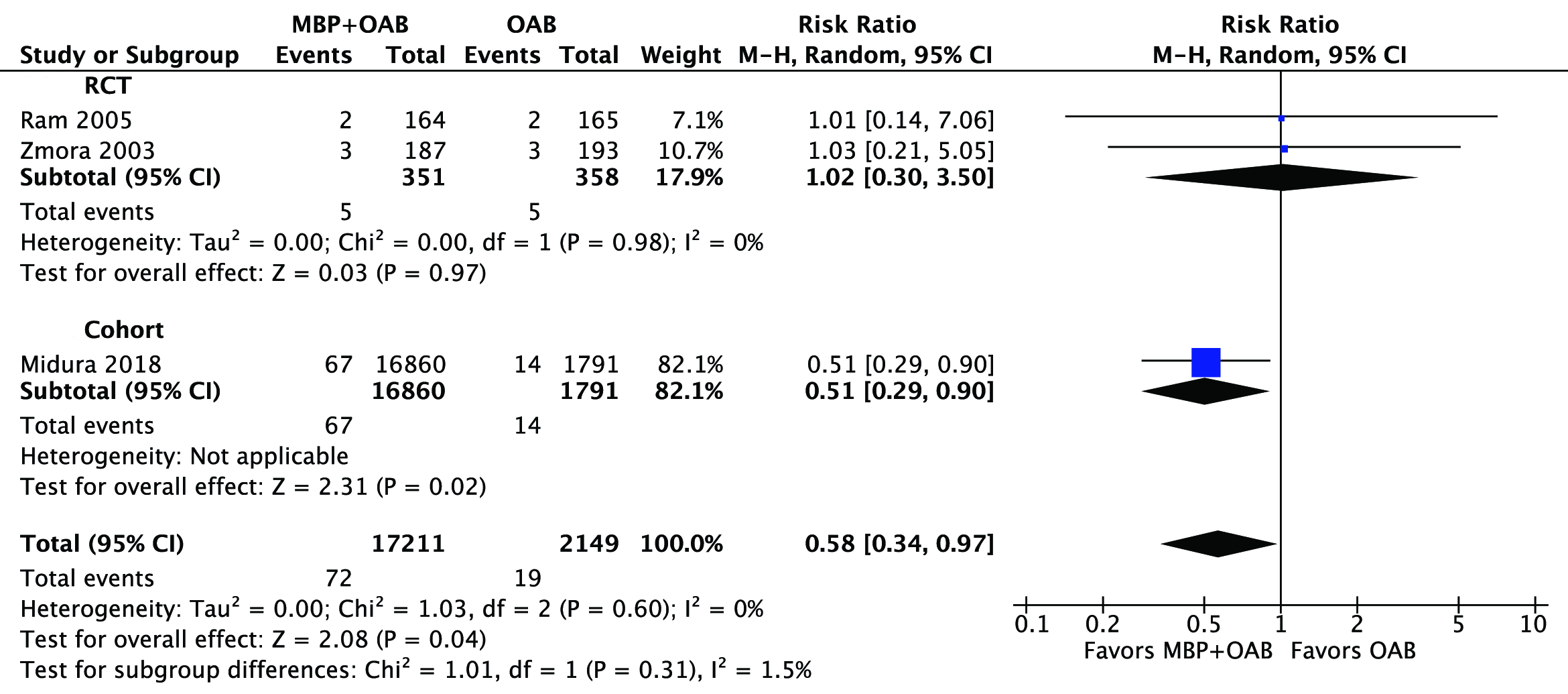


**Supplementary Table 1** – Summary of literature published based upon data obtained from the ACS NSQIP database

| **Author** | **Years of study** | **Study population** | **Outcomes** |
| --- | --- | --- | --- |
| Althumairi *et al*. 2016^1^ | 2012-13 | 19,686 patients  Elective colectomy | Combined MBP+OAB ↓ SSI, anastomotic leak, ileus, sepsis and readmission vs. no prep. |
| Connolly *et al*. 2016^2^ | 2006-14 | 1018 patients  Colorectal resection | SSI reduction strategy (including OAB) resulted in 41% decrease in SSI rates |
| Dolejs *et al*. 2017^3^ | 2012-14 | 4829 patients aged ≥75 years  Elective colectomy | Combined MBP+OAB ↓ anastomotic leak, ileus, SSI and hospital LOS vs. no prep. MBP alone or OAB alone no different to no prep. |
| Garfinkle *et al*. 2017^4^ | 2012-14 | 40,446 patients  Elective colectomy | Combined MBP+OAB significantly ↓ SSI, anastomotic leak, ileus, major morbidity and mortality vs. no prep. No superiority of MBP/OAB vs. OAB alone. OAB alone protective against SSI, AL, ileus, overall morbidity but not mortality rates. |
| Haskins *et al*. 2016^5^ | 2012-13 | 6297 patients  Elective colectomy with primary anastomosis for colon cancer | No difference in severity of anastomotic leak or 30-day mortality by type of preparation used or no prep. |
| Kiran *et al*. 2015^6^ | 2012 | 8442 patients  Elective colorectal resection | Combined MBP+OAB significantly ↓ ileus, anastomotic leak and SSI |
| Klinger *et al*. 2017^7^ | 2012-15 | 27,804 patients  Elective colorectal resection | Combined MBP+OAB significantly ↓ SSI, wound dehiscence and anastomotic leak vs. no prep and lower rate than with OAB alone |
| Midura *et al*. 2018^8^ | 2012-15 | 45,724 patients  Elective colectomy with anastomosis | Combined MBP+OAB associated with significantly ↓ SSI and anastomotic leak. OAB alone associated with significantly ↓ SSI |
| Moghadamyeghaneh *et al*. 2015^9^ | 2012–13 | 5021 patients  Elective colon resection | Combined MBP+OAB in left sided resection significantly ↓ morbidity, SSI, anastomotic leak and intra-abdo collection vs. no prep. MBP or OAB alone no difference vs. no prep. |
| Moghadamyeghaneh *et al*. 2016^10^ | 2012-13 | 27,560 patients  Elective colon resection | OAB administration associated with significant ↓ in incidence of prolonged ileus |
| Morris *et al*. 2015^11^ | 2011-12 | 8415 patients  Colorectal resection | OAB significantly ↓ SSI, hospital LOS and readmission vs. no prep or MBP alone |
| Ohman *et al*. 2017^12^ | 2011-15 | 307 patients  Elective colectomy | Combined MBP+OAB strongest predictor of ↓ SSI *versus* MBP or OAB alone in infection prevention bundle |
| Parthasarathy *et al*. 2017^13^ | 2013 | Overall 17,518 patients undergoing elective colorectal resection with anastomosis, of whom 687 patients developed an anastomotic leak | Preoperative OAB preparation alone associated with reduced risk of anastomotic leak. Preoperative MBP was not associated with anastomotic leak. |
| Parthasarathy *et al*. 2018^14^ | 2015 | 13,959 patients  Colectomy | Preoperative OAB associated with a significantly lower rate of Clostridium difficile infection on univariate analysis, however this significance was lost on multivariate testing. |
| Rencuzogullari *et al*. 2017^15^ | 2012-13 | Overall 10,392 patients aged ≥65 years undergoing elective segmental colectomy with an anastomosis at different levels, of whom 332 patients developed an anastomotic leak | Omitting mechanical bowel preparation and/or preoperative oral antibiotic use were associated with an increased risk of anastomotic leak. |
| Rencuzogullari *et al*. 2017^16^ | 2012-13 | Overall 29,201 patients undergoing elective colectomy, of whom 3834 patients developed postoperative ileus | Omission of OAB pre-operatively associated with significantly increased risk of postoperative ileus |
| Scarborough *et al*. 2015^17^ | 2012 | 4999 patients  Elective colorectal resection | Combined MBP+OAB significantly ↓ SSI, anastomotic leak and procedure-related readmission vs. no prep. MBP or OAB alone no different to no preparation. |
| Schwaartz *et al*. 2017^18^ | 2012-14 | 3679 patients with IBD  Colorectal resection | Combined MBP+OAB significantly ↓ anastomotic leak, ileus, SSI, organ space infection and wound dehiscence |
| Tevis *et al*. 2016^19^ | 2012-13 | 30,101 patients  Non-emergent colectomy | Preoperative bowel preparation protective against anastomotic leak |

MBP=mechanical bowel preparation, OAB-oral antibiotics, SSI=surgical site infection

**References**

1 Althumairi AA, Canner JK, Pawlik TM, et al. Benefits of bowel preparation beyond surgical site infection: a retrospective study. *Ann Surg.* 2016; 264: 1051-1057.

2 Connolly TM, Foppa C, Kazi E, Denoya PI, Bergamaschi R. Impact of a surgical site infection reduction strategy after colorectal resection. *Colorectal Dis.* 2016; 18: 910-918.

3 Dolejs SC, Guzman MJ, Fajardo AD, et al. Bowel preparation is associated with reduced morbidity in elderly patients undergoing elective colectomy. *J Gastrointest Surg.* 2017; 21: 372-379.

4 Garfinkle R, Abou-Khalil J, Morin N, et al. Is there a role for oral antibiotic preparation alone before colorectal surgery? ACS-NSQIP analysis by coarsened exact matching. *Dis Colon Rectum.* 2017; 60: 729-737.

5 Haskins IN, Fleshman JW, Amdur RL, Agarwal S. The impact of bowel preparation on the severity of anastomotic leak in colon cancer patients. *J Surg Oncol.* 2016; 114: 810-813.

6 Kiran RP, Murray AC, Chiuzan C, Estrada D, Forde K. Combined preoperative mechanical bowel preparation with oral antibiotics significantly reduces surgical site infection, anastomotic leak, and ileus after colorectal surgery. *Ann Surg.* 2015; 262: 416-425; discussion 423-415.

7 Klinger AL, Green H, Monlezun DJ, et al. The role of bowel preparation in colorectal surgery: results of the 2012-2015 ACS-NSQIP data. *Ann Surg.* 2017 [Epub ahead of print] doi: 10.1097/SLA.0000000000002568.

8 Midura EF, Jung AD, Hanseman DJ, et al. Combination oral and mechanical bowel preparations decreases complications in both right and left colectomy. *Surgery.* 2018; 163: 528-534.

9 Moghadamyeghaneh Z, Hanna MH, Carmichael JC, et al. Nationwide analysis of outcomes of bowel preparation in colon surgery. *J Am Coll Surg.* 2015; 220: 912-920.

10 Moghadamyeghaneh Z, Hwang GS, Hanna MH, et al. Risk factors for prolonged ileus following colon surgery. *Surg Endosc.* 2016; 30: 603-609.

11 Morris MS, Graham LA, Chu DI, Cannon JA, Hawn MT. Oral antibiotic bowel preparation significantly reduces surgical site infection rates and readmission rates in elective colorectal surgery. *Ann Surg.* 2015; 261: 1034-1040.

12 Ohman KA, Wan L, Guthrie T, et al. Combination of oral antibiotics and mechanical bowel preparation reduces surgical site infection in colorectal surgery. *J Am Coll Surg.* 2017; 225: 465-471.

13 Parthasarathy M, Greensmith M, Bowers D, Groot-Wassink T. Risk factors for anastomotic leakage after colorectal resection: a retrospective analysis of 17 518 patients. *Colorectal Dis.* 2017; 19: 288-298.

14 Parthasarathy M, Bowers D, Groot-Wassink T. Do preoperative oral antibiotics increase Clostridium difficile infection rates? An analysis of 13 959 colectomy patients. *Colorectal Dis.* 2018; 20: 520-528.

15 Rencuzogullari A, Benlice C, Valente M, Abbas MA, Remzi FH, Gorgun E. Predictors of anastomotic leak in elderly patients after colectomy: nomogram-based assessment from the American College of Surgeons National Surgical Quality Program Procedure-Targeted Cohort. *Dis Colon Rectum.* 2017; 60: 527-536.

16 Rencuzogullari A, Benlice C, Costedio M, Remzi FH, Gorgun E. Nomogram-derived prediction of postoperative ileus after colectomy: an assessment from nationwide procedure-targeted cohort. *Am Surg.* 2017; 83: 564-572.

17 Scarborough JE, Mantyh CR, Sun Z, Migaly J. Combined mechanical and oral antibiotic bowel preparation reduces incisional surgical site infection and anastomotic leak rates after elective colorectal resection: an analysis of colectomy-targeted ACS NSQIP. *Ann Surg.* 2015; 262: 331-337.

18 Shwaartz C, Fields AC, Sobrero M, Divino CM. Does bowel preparation for inflammatory bowel disease surgery matter? *Colorectal Dis.* 2017; 19: 832-839.

19 Tevis SE, Carchman EH, Foley EF, Heise CP, Harms BA, Kennedy GD. Does anastomotic leak contribute to high failure-to-rescue rates? *Ann Surg.* 2016; 263: 1148-1151.
